# Modification of Keratin Integrations and the Associated Morphogenesis in Frizzling Chicken Feathers

**DOI:** 10.3390/biology13070464

**Published:** 2024-06-23

**Authors:** Hao Wu, Tsao-Chi Chuang, Wan-Chi Liao, Kai-Jung Chi, Chen-Siang Ng, Hsu-Cheng Cheng, Wen-Tau Juan

**Affiliations:** 1 Graduate Institute of Biomedical Sciences, China Medical University, Taichung 40402, Taiwan; 2 Department of Life Sciences, National Chung Hsing University, Taichung 40227, Taiwan; kjchi@phys.nchu.edu.tw (K.-J.C.);; 3 Department of Biomedical Imaging and Radiological Science, China Medical University, Taichung 40402, Taiwan; 4 The iEGG and Animal Biotechnology Center, National Chung Hsing University, Taichung 40227, Taiwan; 5 Department of Physics, National Chung Hsing University, Taichung 40227, Taiwan; 6 Institute of Molecular and Cellular Biology, National Tsing Hua University, Hsinchu 30013, Taiwan; gcsng15@gmail.com; 7 Department of Medical Research, China Medical University Hospital, Taichung 40447, Taiwan

**Keywords:** frizzling feather, rachis, cortex, medulla, morphogenesis, quantitative morphology field

## Abstract

**Simple Summary:**

Feathers are remarkable skin appendages that serve various ecological functions due to their complex structure and composition. This study focuses on frizzling feathers, which are known for their unique curling patterns. By examining frizzle chickens, both those with two copies of the frizzling gene and those with one, we analyzed how soft and stiff keratin materials are distributed in the shafts of their flight feathers. Our findings show that the way these keratins are arranged affects the internal structure and biomechanics of the feathers, causing them to curl naturally. Specifically, we discovered that a reduction in soft keratin in the core of the feather shaft changes the internal stresses and leads to frizzling. This research enhances our understanding of how curl feather structures develop and adapt to different ecological needs, providing valuable insights into the diversity of feather forms in nature.

**Abstract:**

The morphological and compositional complexities of keratinized components make feathers ingenious skin appendages adapted to diverse ecological needs. Frizzling feathers, characterized by their distinct curling phenotypes, offer a unique model to explore the intricate morphogenesis in developing a keratin-based bioarchitecture over a wide range of morphospace. Here, we investigated the heterogeneous allocation of α- and β-keratins in flight feather shafts of homozygous and heterozygous frizzle chickens by analyzing the medulla–cortex integrations using quantitative morphology characterizations across scales. Our results reveal the intriguing construction of the frizzling feather shaft through the modified medulla development, leading to a perturbed balance of the internal biomechanics and, therefore, introducing the inherent natural frizzling compared to those from wild-type chickens. We elucidate how the localized developmental suppression of the α-keratin in the medulla interferes with the growth of the hierarchical keratin organization by changing the internal stress in the frizzling feather shaft. This research not only offers insights into the morphogenetic origin of the inherent bending of frizzling feathers but also facilitates our in-depth understanding of the developmental strategies toward the diverse integuments adapted for ecological needs.

## 1. Introduction

Feathers represent one of the most intricate functional integuments found on avian skin, characterized by their complex hierarchical branching patterns and superior biomechanical properties [1,2,3,4]. The structural complexity among feathers emerges from the programmed stacking of keratinized components across different scales and regions [5,6]. The multiscale organization of keratin gives rise to a wide range of morphological and material variations [3,7,8,9], each closely linked to specific functional adaptations [10,11,12,13,14]. For example, in sustained flying birds, the flight feathers are supported by resilient shafts that exhibit a polarized distribution of stiff keratin in the dorsal and ventral regions of the rachis cortex, while the medulla is composed of a uniformly high-porosity cellular solid. In contrast, the feather rachis of burst flyers, which must withstand pulsating force loads during wing flapping, features a ridged cortex with a complex geometry and a spatially patterned cellular organization within the medullary core [4]. Therefore, understanding the keratin integration strategies in feathers uncovers the intricated morphogenesis toward a functional biological material.

The feather morphogenesis is highly regulated and orchestrated by an interplay of temporal and spatial mechanisms [1,3,7,15,16,17,18,19,20,21,22,23,24,25,26,27]. Unlike other branching structures observed in nature, the regeneration of a functional three-dimensional feather starts from the distal end, progressing towards the proximal end, and eventually forms a hierarchical appendage anchoring into the skin [1]. Recent molecular studies have identified primary molecular signals that dictate feather development and regeneration through localized expression patterns [28], marking significant milestones in the contemporary research on feather morphogenesis. Furthermore, breakthroughs in understanding how keratins contribute to the intricate structure of feathers [29,30], as well as recent insights into the regional variations in the stacking arrangement of feather keratin fibers [5,6], have deepened our understanding of feather materials through modern methodologies. The superior material features for specific functionalities and the underlying architectural principles of the keratinized feathers have recently been revealed [2,4,31].

Recent advancements in genetic research have provided significant insights into the crucial role of α-keratin genes in the feather structure. The frizzle feather trait, a distinct phenotype characterized by feathers curling outward and upward, has been linked to specific mutations in α-keratin genes. Initial studies identified an 84 base pair deletion in the genomic DNA in the KRT6A (previously known as KRT75) gene [27], involving a 69 bp in-frame deletion in a conserved region of mRNA, leading to a defective rachis and resulting in the frizzled feather phenotype. This mutation disrupts the normal development and structure of the feather rachis, causing the characteristic curling. Further investigations into local chicken breeds have expanded our understanding of this genetic trait. Research on Kirin chickens identified a 15 base pair deletion in the KRT75L4 gene as the causative mutation for frizzle feathers [32]. Similarly, in Xiushui Yellow Chicken, the same 15 bp deletion in KRT75L4 was found to be responsible for this phenotype [33]. These findings, derived from genome-wide association studies (GWASs) and comparative genomic analyses, underscore the significant role of deletions in the KRT6A and KRT75L4 genes in determining the feather morphology.

As the cortical and the medullary components of a feather consist mainly of β- and α-keratins, respectively, the geometrical and mechanical properties of a feather bioarchitecture can be tuned by changing the cortex–medulla integration [4,9,27,34]. Although previous studies have identified the genetic basis of feather frizzling attributed to mutations in α-keratin genes [27], the mechanisms underlying the curling structure remain not fully understood. Considering that the development of feathers with inherent frizzling involves cross-scale modifications of keratin integration resulting in distinct phenotypes, we want to address this gap by uncovering the morphological characteristics of keratinized components in feather rachises under diverse expressions of the frizzle gene. The insights gained from this study not only fill this knowledge gap but may also inspire the fabrication of composite beams with inherent natural bending for various applications.

We leverage the new techniques to resolve the cortex–medulla integration details of flight feathers from chickens exhibiting distinct frizzling phenotypes caused by the mutation of KRT6A [27], as the recent advances in quantitative imaging analyses have revolutionized our interdisciplinary understanding of diversity in feathers [2,4,31]. By examining the morphological combination of the cortex and medulla along the rachis, which retains significant developmental clues from distal to proximal feather regeneration, our aim is to uncover the compositional variations across scales that form different frizzle phenotypes through a series of quantitative analyses.

## 2. Materials and Methods

**Figure 1:**
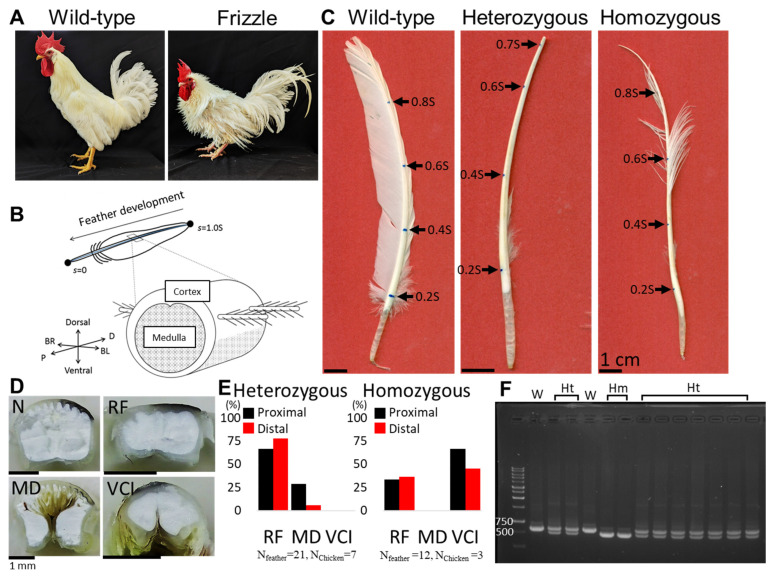
Phenotypes of frizzle chickens and the frizzling feathers. (**A**) The pictures of the domestic wild-type (**left**) and the frizzle (**right**) chickens. (**B**) The definitions of the coordinates describe the relative position along the rachis (**upper panel**) and the coordinates and components of the feather rachis shaft (**lower panel**). (**C**) The scanning images of the flight feathers collected from the wild-type chicken (**left**), and heterozygous (**middle**) and homozygous (**right**) frizzle chickens show three different phenotypes. Blue dots marked on the dorsal surface of the rachis denote the rachis coordinate *s* corresponding to different positions (indicated by arrows) along a feather shaft using full accumulated length S as the unit from proximal (*s* = 0) to distal (*s* = 1.0S). For details of tracing *s*, see Figure 2A. (**D**) Examples of morphological features found in frizzle chicken rachis (N: normal; RF: rachis flattening; MD: medullary cell depletion; VCI: ventral cortex invasion). (**E**) Occurrence probability of the specific morphological feature on the proximal and distal portions of rachis, sampled across multiple heterozygous and homozygous frizzle chicken feathers. N_feather_ and N_chicken_ refer to the number of sampling feathers and chickens, respectively. (**F**) PCR products were amplified from the genomic DNA of phenotypically normal (W), heterozygous (Ht), and homozygous (Hm) frizzle chickens. The mutant allele of KRT6A (previously named KRT75) exhibits an 84 bp deletion in genomic DNA, resulting in a smaller size compared to the wild-type allele. This figure shows one of the gel electrophoresis results from our genotyping analysis.

Feather sample collection. We chose the chicken flight feather as the model specimen for our study. Within avian research, domestic chickens, particularly frizzling chickens, as shown in Figure 1A, have emerged as invaluable model organisms due to the recent comprehensive research across disciplines [27,35,36,37,38,39,40,41,42,43,44,45]. Flight feathers were obtained from the primaries and secondaries of the wing from 13 mature Leghorn chickens representing three phenotypic traits: wild-type (3 chickens, as control group), homozygous (3 chickens), and heterozygous (7 chickens) for the frizzle gene. Feathers were carefully plucked, cleaned with wet tissue paper (soaked with distilled water or 75% ethanol), and air-dried to ensure their integrity for subsequent analyses. The genotypes were determined by polymerase chain reaction (PCR). Genomic DNA was extracted from feather follicles using the DNeasy Blood & Tissue Kit (Qiagen, Hilden, Germany). The region encompassing the KRT6A gene (NCBI accession no. NM_001001313, previously known as KRT75) was amplified via PCR using specific primers (forward: 5′-TTTCTTCTTTCCCTCCCACT-3′, reverse: 5′-GTTCTGCTTCCCCTGATTAT-3′), as described previously [27].

**Figure 2:**
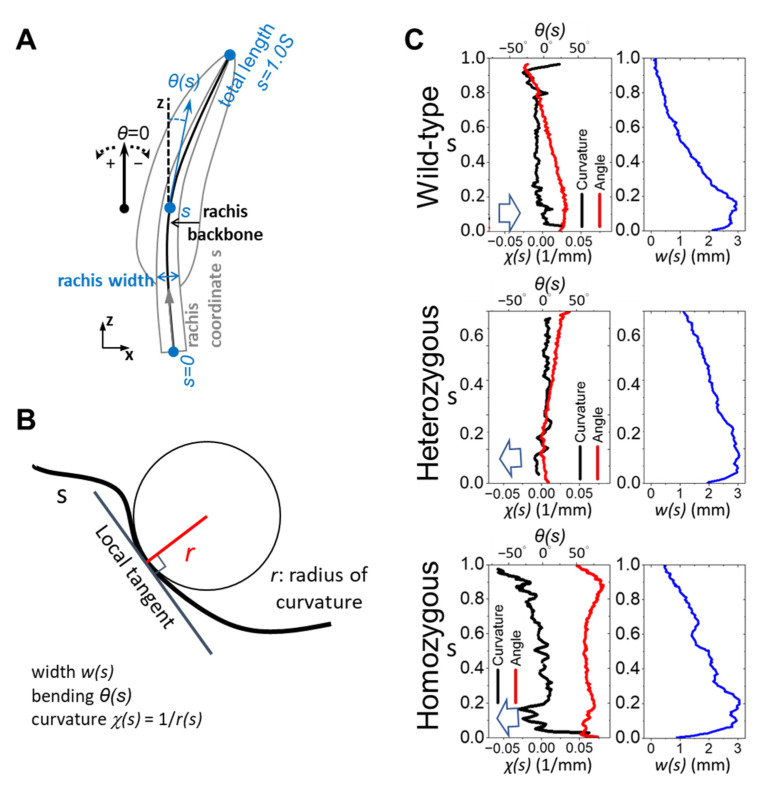
Quantifying the bending of feather shafts. (**A**) The rachis backbone is obtained by tracing the mid-point between opposite edges of the rachis contour along the feather shaft. Since the feather shaft is composed of the barb-free proximal calamus tube, as well as the barb anchoring rachis above the skin, we define the rachis coordinate *s* from the proximal end of the calamus as *s* = 0 and the distal end of the feather as *s* = 1.0S, where S is the accumulated full feather length. The local deflection angle *θ*(*s*) and curvature *χ*(*s*) are defined in (**B**), and the rachis width *w*(*s*) along *s* can be characterized by analyzing the digital image, as shown in the red, black, and blue curves in (**C**), respectively. The inset arrow in (**C**) points toward the corresponding dorsal direction in each panel.

Full-length feather morphology digitizing and characterization. The whole feather morphology projected on the two-dimensional plane was quantified by reading the coordinates of contour pixels from high-resolution images captured by a flatbed scanner. To describe the spatial structure of feathers, we established a coordinate system centered on the feather (Figure 1B). This system defines three orthogonal axes: Proximal–Distal (P–D), Dorsal–Ventral (D–V), and BarbL–BarbR (BL–BR). Note that, in a typical flight feather, the leading vane against the airflow is narrower than the trailing one. The P–D axis aligns with the curving feather shaft, the D–V axis delineates the outer and inner sides of the shaft, and the BL–BR axis represents the left and right sides when viewed from the distal end towards the proximal end. A custom MATLAB algorithm was developed to quantitatively characterize the curly rachis and identify the backbone trace of a rachis as the rachis coordinates *s*. Measuring parameters such as total feather length (S), local width, deflecting angle *θ*, and curvature of the feather shaft *χ* as functions of *s* can be carried out (Figure 2A,B). 

Sectioning feather shaft for cross-sectional analyses. Feather shafts were physically sectioned to analyze their internal structure cross-sectionally. Samples were cut into segments, embedded in paraffin blocks, and sliced perpendicularly to the rachis axis across the cross-section. Sections were obtained regularly at a fixed interval of 5 μm along the shaft, and care was taken to preserve their orientation and integrity. In practice, we performed continuous sectioning of rachis samples to statistically sample and analyze the rachis cross-sectional morphology [46]. Paraffin-embedded feather shaft samples were continuously sectioned into slices approximately 5 μm thick. Sections were mounted on glass slides for microscopy investigation, ensuring a sequential arrangement from the distal to proximal order. After sectioning, the remaining portions of the rachis embedded in paraffin blocks were used for cortical morphology investigation at corresponding positions.

Optical microimaging of rachis cross-section. Feather shaft sections were imaged using Olympus BX51 optical microscopy (Olympus, Tokyo, Japan) to visualize their internal structure. Cortical and medullary morphology were recorded using reflected and transmitted light, respectively. Due to the limitation of the view field, images were stitched together to obtain comprehensive two-dimensional representations at high resolution. Further quantitative analyses of the morphology of the cortex–medulla integration, as well as the cellular morphology within the medulla region were then performed.

**Figure 3:**
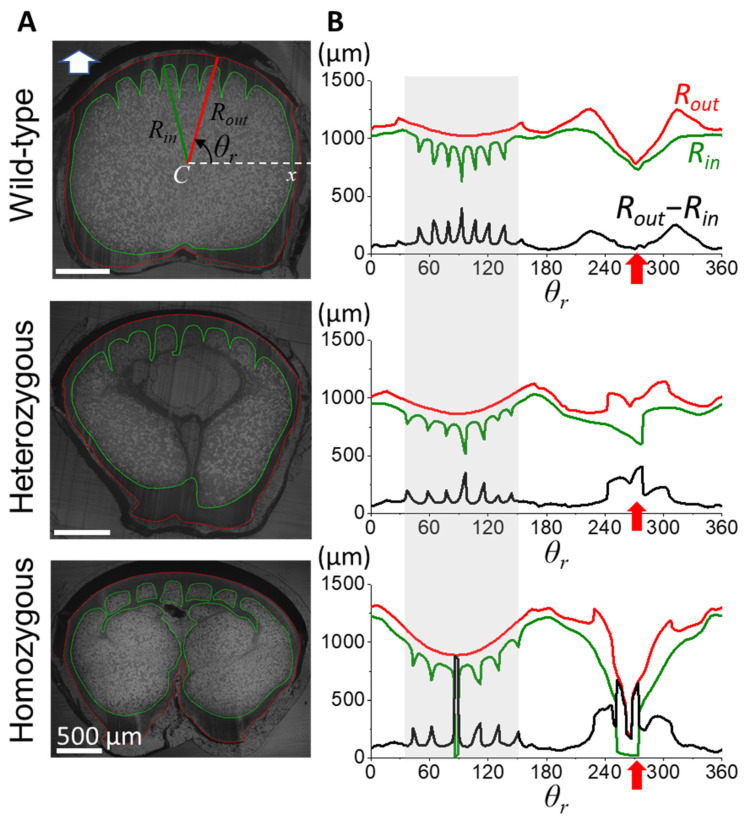
Diverse cross-sectional morphology in the proximal flight feather rachis among different phenotype frizzle chickens. (**A**) The cross-sectional microimages at 0.4S of the flight feather rachises are taken under an optical microscope. The insert arrow on the top-left corner of the microimage indicates the dorsal direction of the rachis. The outer and inner contours of the cortex are marked by the red and green loops, respectively. The inner cortex contour is also the cortex–medulla interface. We define the radiuses of outer and inner radiuses as *R_out_* and *R_in_*, respectively, measuring from the geometric center *C* of the rachis cross-section, to characterize the variation of cortex thickness (*R_out_*-*R_in_*) along the azimuthal (*θ_r_*) direction around the rachis. (**B**) The measured outer and inner radiuses as *R_out_* (red) and *R_in_* (green), and the calculated cortex thickness *R_out_*-*R_in_* (black) along the azimuthal (*θ*) direction. Gray shadow regions marked the angular distribution of the dorsal cortex. The red arrow of each panel indicates the location of the ventral groove in each measurement.

The cortex morphology, described by the profile between the outer cortex surface and inner medulla contour represented by red and green loops, respectively (Figure 3A), was quantified using azimuth angles to describe variations in the cortex’s shape around the shaft’s cross-section. MATLAB algorithms (MathWorks, Natick, USA) were employed to delineate cortical contours and calculate cortical thickness (*R_out_*-*R_in_*) across the range of azimuth angle (*θ_r_*), as illustrated in the cross-sectional image of wild-type flight feather cross-section in the result figure. Medullary morphology was quantified by analyzing the porous structure of the medulla using MATLAB image-processing algorithms, termed quantitative morphology field (QMorF) [4,46], which allows direct visualization of the medulla’s cellular morphology in three-dimensional spatial distribution. Binary images of the medullary reticulum were generated, and properties of individual pores were extracted. These properties were used to calculate scalar fields representing the distribution of cellular morphology in medulla, visualized using color heatmaps [4,46].

Histology and fluorescent staining of developing feather. A primary flight feather from a wild-type Leghorn chicken’s wing was plucked and allowed to regrow to half of its original length. The growing feather follicle was collected and cut according to the desired developing stages. The cut feather follicle samples were treated with 4% paraformaldehyde–PBS (phosphate-buffered saline) solution overnight at 4 °C, dehydrated with ethanol series, and embedded in paraffin for further sectioning. Sections that were 5 μm thick were prepared cross-sectionally from the paraffin-embedded samples along the proximal–distal axis of the feather. The sections were dewaxed with Xylene for 15 min twice, then rehydrated with an ethanol series to PBS. The rehydrated sections were treated with citric buffer (10 mM citric acid, 0.1% NP-40, and pH 6.0) at 95 °C for 30 min and washed off with PBS for 5 min three times. Afterward, the sections were stained with DAPI and mounted using Fluoromount Aqueous Mounting Medium (Sigma-Aldrich, St. Louis, MO, USA). Fluorescent images of the sections were taken by a Nexcope NCF950 laser confocal microscope (Ningbo Yongxin Optics, Ningbo, China).

## 3. Results

### 3.1. Genotyping and Phenotyping of Frizzle Flight Feathers

The appearance of flight feather morphology exhibits distinct differences among the three phenotypes: wild-type, heterozygous frizzle, and homozygous frizzle chickens. Frizzle chickens exhibited these distinctive feather phenotypes based on their genotypes of KRT6A (previously named KRT75): homozygous individuals carried two mutant alleles, while heterozygous individuals carried a single mutant allele (as shown in Figure 1F). The wild-type chicken flight feather showing a normal branching architecture serves as our reference for identifying the morphological changes under the influences of the frizzling gene expression. In the flight feather rachis of frizzle chickens, intriguing morphological features, such as the rachis flattening, medulla depletion, and ventral cortex invasion, were observed (Figure 1D). The occurrence probabilities of these morphological features were displayed in Figure 1E. We then chose a feather featuring the primary morphological characteristics of each category as a representative for the detailed analyses (Figure 1C). In wild-type chickens, as shown in the left panel of Figure 1C, a typical flight feather comprises the bilaterally asymmetric lamellar vane and a naturally persistent bending central rachis shaft toward the trailing side. Conversely, flight feathers from heterozygous and homozygous frizzle chickens, as shown in the middle and right panels in Figure 1C, respectively, exhibit sparse lamellar vane structures due to the loss of barbs. Additionally, irregular bending and width variations are found in the frizzle feather rachises.

### 3.2. Full-Length Characterization of the Frizzling Feather Shaft

To quantitatively describe the spatially varied rachis bending, we analyzed the local deflection angle *θ*, curvature *χ*, and width *w* by tracing the backbone of the rachis along the rachis coordinates *s*, as illustrated in Figure 2A,B, among the three feather phenotypes. Red curves in Figure 2C show the local deflection angle *θ* of the rachis backbone along the coordinates of the three representing feathers in Figure 1C, respectively. Despite the initial calamus-to-rachis transition region of the feather shaft at the very proximal end (*s* < 0.2S), the wild-type flight feather rachis shows a persistent bending toward the ventral side, hence the stable curvature over a wide range from 0.2S to 0.75S. 

An important deflection associated with an abrupt curvature fluctuation appears at the distal end after 0.75S. Overall, the rachis width of the wild-type chickens exhibits exponential-like decay toward the distal direction, suggesting a gradual tapering of the rachis from the proximal to distal regions. Compared to wild-type feathers, the rachis structure of frizzle feathers displayed deviations from the wild-type pattern, exhibiting a fluctuating shaft curvature and width. As the defection and curvature curves look similar to the wild-type case, the tapering of the heterozygous frizzle feather between 0.2S and 0.6S is less aggressive. It is interesting to note that the homozygous frizzle shows multiple irregular deflections and, therefore, curvature fluctuations along *s*. It even exhibits a dorsal-ward bending near the distal end, as reported in previous literature [27]. The rachis width variation of a homozygous frizzle feather shows certain irregularities around 0.3S and 0.6S. 

### 3.3. Morphological Characterization of Cortex from the Rachis Cross-Section

The morphology of the stiff and dense cortex is crucial in determining the structural integrity and biomechanical properties of feathers. To investigate the cortical morphology of flight feather rachis in wild-type chickens, as well as the heterozygous and homozygous frizzle chickens, we traced the radiuses of the outer (*R_out_*, red loop) and inner (*R_in_*, green loop) contours of the cortical region with respect to the geometrical center C, and then calculated the cortex thickness (*R_out_*-*R_in_*) azimuthally along the polar coordinate *θ_r_* from the cross-sectional images at different positions of the shaft in Figure 3. We chose positions 0.4S and 0.6S as the representative sites of the proximal and distal rachis, as the midpoint of the rachis is usually the transitional portion for the rachis morphology.

We observed a distinct cortex–medulla integration in the proximal half of the flight feather rachis according to the frizzling phenotype. In the position at *s* = 0.4S, the cross-section of the wild-type rachis (upper panel of Figure 3A) shows a typical proximal rachis configuration of a chicken flight feather [4] with a porous medulla core (the region enclosed by the green contour) surrounded by the outer cortical layer (the region between red and green contours). The internal side of the rounded dorsal cortex (*θ* between 45–135°, gray shadow region in Figure 3B) shows the periodically arranged seven cortical ridges, which are bilaterally distributed about the mid-line (see seven spikes in the *R_out_*-*R_in_* curve distributed around *θ* = 90°). The ventral groove exhibited a central depression with a relatively smooth cortical thickness variation (indicated by the red arrow). In heterozygous frizzle chickens (the middle panel of Figure 3), the internal cortex–medulla integration deviates from the typical wild-type configuration, showing slight irregularities in the arrangement and thickness of the cortical ridges in the dorsal region. Additionally, an increased cortical thickness was found around the ventral groove compared to the wild-type case.

In the lower panels, we even discovered a more complicated proximal rachis composition of the homozygous frizzle chickens compared to the previous two cases. Unlike the very distinct cortex–medulla regions in the outer shell and inner core of the rachis beam among flight feathers, in the microimage, the tips of the dorsal ridges are laterally connected with the dense cortical material and contribute to the isolated inter-ridge medulla chambers. The formation of the intersecting cortical layer within the core region of the rachis contributes to the artifacts of the dorsal cortex thickness within *θ* = 85° to 90°. In addition, a strong invasion of the ventral cortex is also observed, causing an increased cortical thickness near the ventral groove region. This observation is an example of the occurrence of ventral cortical invasion in homozygous chickens, as previously reported in Figure 1E.

**Figure 4:**
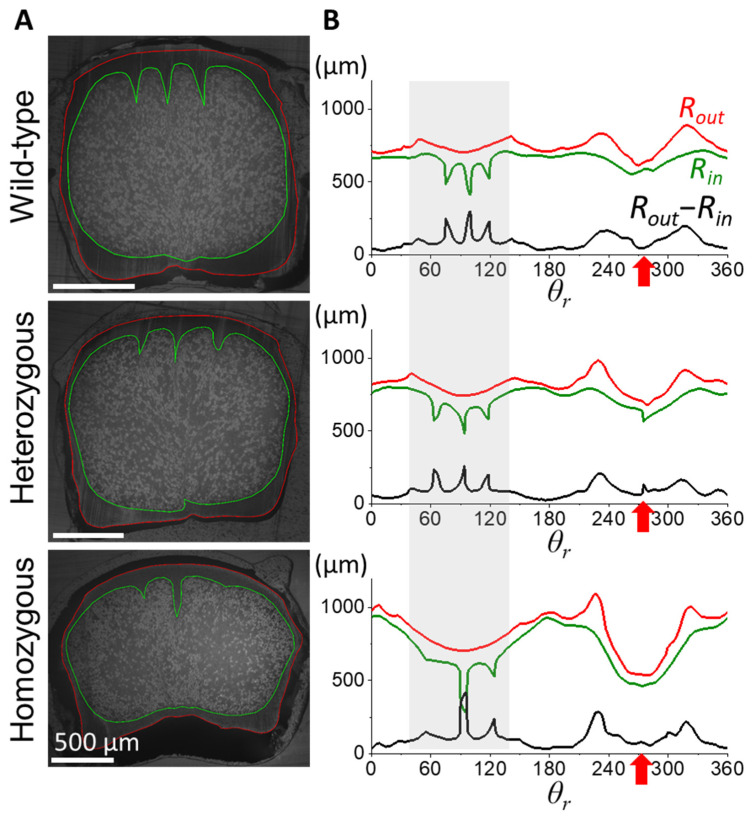
Comparison of cross-sectional morphologies in the distal half of flight feather rachises among different phenotype frizzle chickens. (**A**) The cross-sectional images at 0.6S of the flight feather rachises are taken under an optical microscope. (**B**) The measuring traces of the outer and inner radiuses as *R_out_* (red) and *R_in_* (green), and the calculated cortex thickness *R_out_*-*R_in_* (black) along the azimuthal (*θ*) direction. Gray shadow regions marked the angular range of the dorsal cortex. The red arrow indicates the angular position of the corresponding ventral groove in each case.

In contrast to the distinctive cortex–medulla integration in the proximal half of the rachis, the distal half of the rachis shows subtle differences among frizzling phenotypes, as demonstrated in Figure 4. At the 0.6S cross-section, the cross-sectional geometry of wild-type rachis maintained a characteristic rounded rectangular shape, with three similar-sized cortical ridges arranged around the mid-dorsal. The central depression of the ventral groove was evident, accompanied by a localized slight cortical thickness change. In heterozygous frizzle chickens, the cortical cross-section at 0.6S closely resembled that of wild-type chickens, displaying a rounded rectangular shape with symmetrically arranged dorsal cortical ridges. However, the two laterally outer ridges are statistically smaller and slightly distorted compared to the central one. We also found a small-degree discontinuity of the contour, resulting in the tiny spike of the (*R_out_*-*R_in_*) around the ventral groove. The rachis cross-section of the homozygous frizzle chickens also exhibits a rounded rectangular cortical cross-section but seems more laterally flattened compared to the previous two cases. Although dorsal cortical ridges still exist, their spatial ordering and symmetry are severely disturbed. The fluctuating cortical thickness around the ventral groove region is easily seen.

### 3.4. Quantitative Morphology of Cellular Structures in Rachis Medulla

The analysis of cortex morphology along the feather shafts revealed differences in cortical structures in both heterozygous and homozygous frizzle chickens from the typical wild-type case. We applied the innovative quantitative morphology field (QMorF) analysis [4] to unravel the morphology of the cellular structure from the cross-sectional micro-images of the feather shaft medulla. We particularly focused on the positions at 0.4S and 0.6S in the rachis coordinate, for which we conducted thorough cortex morphology analyses showing severe configurational diversities in previous sections.

**Figure 5:**
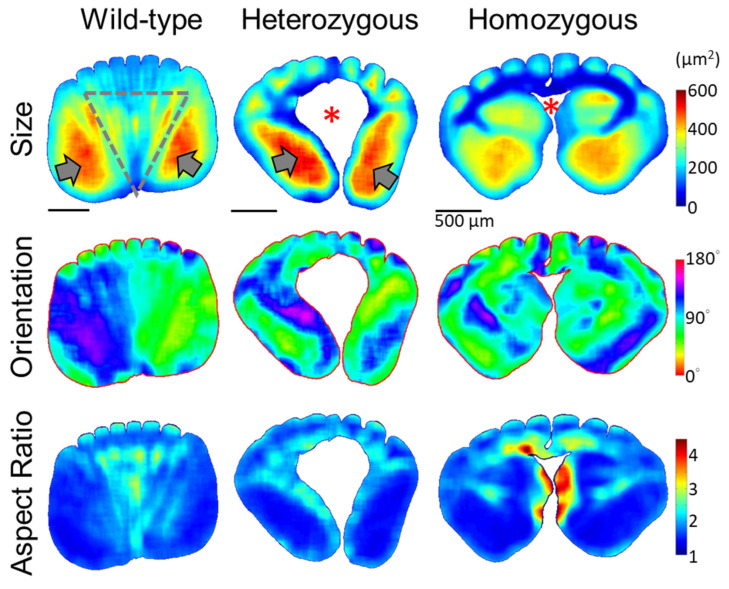
Quantitative morphology field (QMorF) analyses of the cross-sectional cellular structure in the rachis medulla at the proximal half (0.4S) of the flight feathers. The coarse-grain averaging of the quantified morphology of local cellular pores from the microimage of the medulla cross-section reveals the distributions of the cellular morphology features, such as size, orientation, and aspect ratio (from top to bottom), over the entire cross-sectional area of the rachis medulla. The gray arrows indicate the large-cell-size zones on opposite lateral sides of the medulla. A gray dot triangle marked the fan-like region with periodically arranged cell bands along the D–V direction, showing alternative colors laterally. The red stars in the signal-free “size” QMorF plots of the heterozygous and homozygous cases indicate the cell depletion zone in the frizzling feather medulla.

From the heatmaps representing the distributions of cellular morphology characteristics, such as the size, orientation, and aspect ratio of cellular cross-sections in Figure 5, the features of medullary cells revealed by the QMorF show dramatic spatial features at various levels. In the flight feather rachis of the wild-type chicken, the contour of the medulla region exhibited a slightly flattened rectangular shape with rounded corners. According to the heatmap patterns, morphological features of the medullary cells are, in general, bilaterally symmetric. Equidistant sharp depressions of the contour on the dorsal side, aligned with the invasion of dorsal cortical ridges, are in contrast with a wide and shallow depression on the ventral side. Cells on the opposite lateral sides show two distinct zones composed of relatively large cells (reddish color in the size heatmap) with their aspect ratio close to 1 (blueish color in the aspect ratio heatmap). Within the fan-like region between these two large-cell zones, shrinking from dorsal to ventral as marked by the gray dot triangle in Figure 5, striping patterns exist from a wide lateral span of the dorsal region and converge to the ventral depression around the mid-line. The striping pattern exists among all cellular morphology characteristics, i.e., size, orientation, and aspect ratio, and the stripe arrangement exhibits periodicity laterally. The coherences of cell size, orientation, and aspect ratio suggest that keratinized cells in the medulla experienced a highly ordered morphological banding. We termed these striping patterns as cell bands reflecting the undulation of the biomechanical field to collectively fold the medullary cells in a spatially organized way during the rachis morphogenesis [4].

In the heterozygous frizzle chickens (Figure 5, mid-column), the medulla profiles exhibit distinct features compared to the wild-type case at the same 0.4S region. A flattened oval shape with multiple equidistant depressions on the dorsal side characterizes the medulla contour. A noticeable cell depletion zone in a bulb shape, which has no keratinized medullary cell to retrieve the QMorF signal, occupying the core of the medulla is characterized. Such a medullary cell depletion is usually found in the proximal portion of the heterogeneous samples, as reported in Figure 1E. Elongated cells tilting toward the corresponding dorsal–lateral directions on each side were observed around the cell depletion zone on the ventral side. The cell depletion zone seems to interrupt the periodic cellular stripes extending from the medullary ridge and leaves only localized patches of cells with similar morphological features arranged between the invasions of neighboring cortical ridges. From the coarse-grain pattern of the QMorF, the medulla structure of the heterozygous frizzle chicken feather can be considered as a medulla of the wild-type case after removing the fan-like cell-band zone.

The rachis medulla of the homozygous frizzle chickens in the right column of Figure 5, at first glance, seems a combination of features of the wild-type and heterozygous cases. However, detailed inspections suggest that the medulla configuration could be a stronger developmental modification beyond the heterozygous case. In the homozygous case, the medulla contour is characterized by a shell-like profile with a wider dorsal and a shrunk ventral side, and, hence, a more flattened contour. Similar to the QMorF pattern of the heterozygous case, a notable absence of a fan-like striping pattern between the medullary ridges was observed, replaced instead by a group of small, elongated cells extending from the ventral depression to the center of the medulla (as evidenced by the size and aspect ratio heatmaps). The elongated small cells then arrange laterally and eventually form a T-shaped pattern around a central cell-depletion zone. The presence of a T-shaped small-cell cluster, starting from the mid-ventral region along with the central void and then bilaterally spanned, intersects the whole medulla structure and disrupts the typical formation of periodic stripe patterns. 

**Figure 6:**
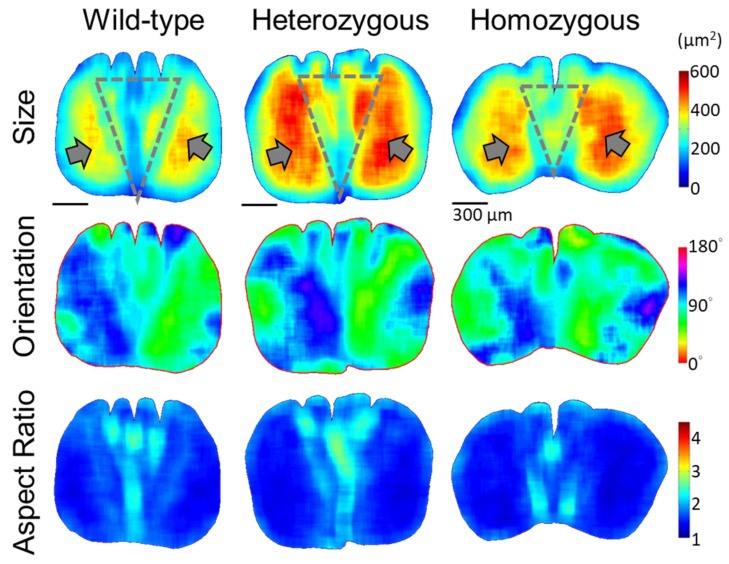
Quantitative morphology field analyses of the cross-sectional cellular structure in the rachis medulla at the distal half (0.6S) of the flight feathers. The quantitative morphology distributions of the medullary cells from the cross-sectional images at 0.6S, such as size, orientation, and aspect ratio, are shown from top to bottom, respectively. The gray arrows indicate the large-cell-size zones on opposite lateral sides of the medulla. Gray dot triangles mark the fan-like region with periodically arranged cell bands along the dorsal–ventral direction, showing alternative colors laterally. No cell depletion zone is found within the medulla of the distal half of the rachis.

In the distal half of the rachis, the disruption of the medulla structures exhibits minor distinctions among flight feathers between wild-type and frizzle chickens (Figure 6). In the position around 0.6S, the rachis medulla of the wild-type chicken appears as a rounded square with rounded corners, featuring equidistant depressions on the dorsal side corresponding to dorsal medullary ridges. Heterozygous frizzle chickens display similar rachis medulla profiles, with only slightly distorted depressions and periodic arrangements of elongated cells. The medulla of homozygous frizzle chickens presents a flatter oval shape, with variations in the depth of the depressions compared to the other phenotypes. As the medulla contours are slightly changed, the QMorF patterns among these three cases all show the simple bilateral symmetric feature and preserve one center cell band along the D–V direction near the mid-line of the medulla.

**Figure 7:**
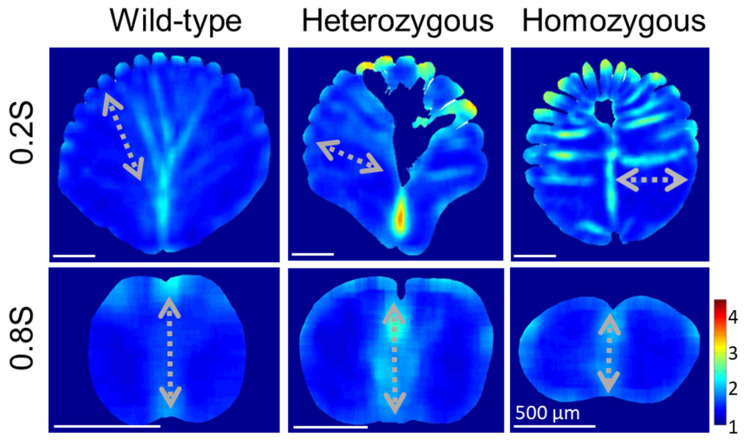
Quantitative morphology field analyses of the cross-sectional cellular aspect ratio in the rachis medulla at the proximal (0.2S) and distal (0.8S) parts of the flight feathers. Laterally arranged elongated cells form horizontal strips from cortical ridges toward the deeply invasive cortex from the mid-ventral region in the proximal parts of the frizzling feather rachis. The gray dash arrow in each panel indicates the representative orientation of the cell band in each cross-section.

To identify the trend of QMorF patterns along the full rachis, we further explored the proximal and distal ends of the rachis while the ratio of area portions between the medulla and cortex decreases as *s* increases. We performed QMorF analyses of the proximal and distal portions of the rachis medulla at 0.2S and 0.8S, respectively, to compare with the results from the middle rachis at 0.4S and 0.6S, as shown in Figure 5 and Figure 6. Demonstrated by the aspect ratio heatmaps in Figure 7, the proximal rachis at 0.2S of the wild-type chicken flight feather displayed a circular shell-like medulla profile with equidistantly spaced medullary ridges on the dorsal side and a singular depression on the ventral groove. The presence of periodic stripe patterns, resembling leaf veins, indicated a symmetrical fan-shaped arrangement of medulla cells, extending from periodically arranged dorsal ridges to the ventral groove. This morphological pattern is consistent with observations at 0.6S and 0.4S but is more pronounced at the more proximal 0.2S.

The rachis medulla of the heterozygous chicken feather also exhibits a shell-like medulla morphology with a considerable cell depletion area at the 0.2S section, albeit with a narrower ventral width and irregularly spaced dorsal depressions. The presence of a distinct cell-depletion zone on the dorsal side disrupted the periodic fan-shaped stripe patterns within the medulla, affecting the cell orientation distribution and persistency. In the case of homozygous frizzle chicken, periodic stripe patterns are arranged mainly along the lateral direction toward the deeply invasive ventral cortex in the cross-sectional mid-line. On the contrary, in the position near the distal rachis at 0.8S, the contours of the medulla cross-sections are flattening according to the frizzling degrees, from wild-type to heterozygous to homozygous frizzling expression. However, the bilateral symmetric distributions of the cellular aspect ratio with a group of elongated cells occupying the mid-line region of the medulla are simultaneously found among three phenotypes.

**Figure 8:**
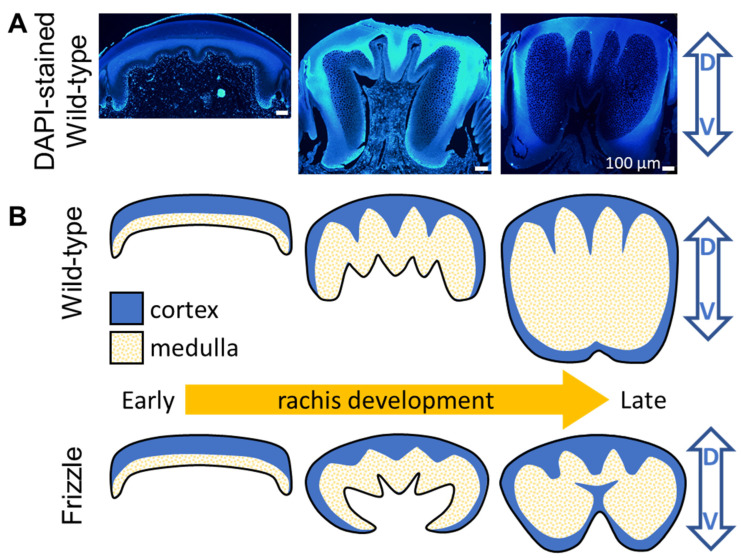
The rachis morphogenesis for different frizzling phenotypes. (**A**) The fluorescent (DAPI) images sample from the different stages of a developing feather shows the dorsal to ventral rachis morphogenesis. (**B**) The hypothetical sketches of the morphogenetic process of normal (**upper**) and frizzling (**lower**) feather rachises. For frizzling feather, the depression of the α-keratin dominated medullary cells in ventral area should provide weak mechanical support for the development of thick rachis and cause the ventral invasion from lateral cortical material toward the medullary core. The D-V arrows indicate the dorsal-ventral direction of the rachis.

To understand the morphogenesis of the cortex–medulla integration in a rachis, we investigated the cross-section of a developing wild-type flight feather within a follicle to visualize the morphogenetic process. From the fluorescent (DAPI) microimages sampled at different stages of feather development in Figure 8A, we observed the progressive dorsal-to-ventral morphogenesis of the rachis. Initially, we saw a dorsal-cortex-dominated configuration. This transitioned to an intermediate stage where a developed dorsal–lateral cortex is associated with a thick layer of medullary cells proliferating toward the rachis core. Finally, we observed a ready-to-close ventral cortex with a nearly fully filled medulla, as shown from left to right in Figure 8A. These observations are summarized in the schematic of the rachis morphogenesis process in the upper panel of Figure 8B. 

## 4. Discussion

This study employs a comprehensive approach to unraveling the morphological changes observed in feather shafts of chickens exhibiting different phenotypes. Using image-based analyses, we systematically quantified the structural features of feather shafts across scales and elucidated the underlying constructing mechanisms driving the variations in the frizzling feather. Considerable morphological differences, particularly in the ventral region of the rachis, among flight feathers from wild-type, homozygous, and heterozygous frizzle chickens are observed. 

Since the rachis is a natural composite beam of the outer cortex shell and the inner medulla core [2,4,47], investigating the cross-sectional cortex–medulla integration reveals the distinct constructions of feather shafts for distinct phenotypes. The rachis of the wild-type feather rachis is integrated in a highly organized way throughout morphological features, such as the symmetric and periodic distribution of the regional cortex structure and patterned cellular morphologies in the medulla. The cortex ridges on the dorsal and ventral sides of the rachis contribute to the material reinforcement against the bending and, therefore, maintain the overall stability of the shaft [4,6]. Additionally, the medulla displayed a highly organized distribution of cells, forming characteristic periodic patterns extending from the dorsal cortex ridges to the ventral groove, suggesting a well-programmed mechanical field sculping the light and resilient cortex–medulla composite beam during the harmonic rachis morphogenesis [4]. Frizzling feathers exhibited drastic morphological changes mainly in the ventral region of the rachis, particularly in the later stages of proximal feather growth. The cortex in this region displayed irregular thickness variations and distortions, which may cause irregular material allocation along the rachis.

The rachis, as the structural backbone to mechanically support the feather branches, contributes the primary biomechanical element for introducing the bending and frizzling of a feather. The smooth curvature of a normal flight feather is produced by continuous and balanced mechanical forces influenced by some key factors, including the accumulation of keratin material within the feather follicle. The perturbation of keratin accumulation modifies the distribution of the material and the associated internal stress of the cortex–medulla complex along the composite feather shaft, resulting in the localized deflections. In the ventral medulla in a frizzling feather rachis, there is a severely different configuration compared to the wild-type case, which seemingly correlates with the mutated keratin gene expression pattern during feather growth [27]. As the soft keratinized cells in the medulla can be considered the scaffold for stacking the stiff β-keratin of the cortical shell during the construction of a feather architecture [9], the misexpression of the medullary keratin may lead to insufficient scaffolding materials. The morphological perturbations of the cross-sectional rachis integration, such as the reduction in the rachis thickness, invasion of the ventral cortex, intersecting of the fan-like cell band region, and distortion of the dorsal cortical ridge, make the frizzling feather composite significantly different from the typical wild-type flight feather as presented in Figure 1D,E. For a thin barb thread of the feather branch, which consists of only a limited portion of the supporting medulla [47], the deficiency of medullary cells could lead to a decrease in structural strength during morphogenesis. This deficiency ultimately results in the exhibition of a sparse lamellar vane structure in frizzle feathers. Considering the structural complexity, the making of a frizzling feather should be more diverse and intricate than other skin appendages with simpler structures, such as curly wool [48]. The off-balance of local material integration could also introduce peculiar morphological patterns at small scales. In Figure 5, the presence of a T-shaped small-cell cluster in the rachis medulla of the homozygous frizzle chickens suggests that the medullary cells not only experienced an invasive biomechanical field toward the dorsal but also turned into a lateral arrangement, presumably under the influence of horizontal mechanical components. This contributes two opposite swirling QMorF patterns on the opposite sides of the medulla, both streaming from the mid-ventral region. 

These findings underscore the dynamic interplay between the cortical and medullary structures in shaping the curly feather shafts. As the cell band revealed by QMorF suggests the architectural details of the dorsal to ventral keratin integration toward a typical rachis, a highly localized perturbation of the developmental blueprint, such as the discovered modification of keratin integration, even on the soft, flexible α-keratin in the ventral rachis medulla, dramatically alters the morphogenetic production of a natural composite and significantly contributes to the biodiversity of feathers for the adaptation [9,27]. Therefore, the diverse cortex–medulla integration in the proximal rachis of the flight feather with different frizzling degrees suggests the distinct perturbation levels of the typical cortical development of the frizzling feather morphogenesis. These morphological perturbations among categories further suggest that the expression of the frizzling gene alleles morphogenetically alters the original construction of the flight feather rachis, hence the curled feather phenotypes. Hence, in Figure 1E, the medullary cell depletion, simply due to the lower expression of α-keratin, is often observed in the heterozygous case; the ventral cortex invasion, due to the non-scaffolding cortex development under the severe depression of α-keratin, is mainly found in the homozygous case. As previously discussed, since the α-keratin-dominated medulla plays a vital structural core in supporting the rachis morphogenesis, our quantitative investigation of the medulla’s cellular morphology across scales reveals the constructing process behind the curling cortex–medulla composite. Combining the progressive dorsal-to-ventral development of the rachis in Figure 8A and recent genetic and developmental pieces of evidence [27,34], distinct rachis-morphing models of the wild-type and frizzling feather rachises through modifying the ventral medulla proliferation can be illustrated by the conceptual plot in Figure 8B. While our previous study and this work provide quantitative evidence linking genetic mutations in the KRT6A (previously named KRT75) frizzle gene [27,34] to morphological changes in feather shafts, the further analysis of smaller structural variations, such as the changes in keratin fiber arrangement at the sub-μm scale, may provide deeper insights into the effects of variations and the biological design on feather microstructure.

Inspired by the inherent bending observed in frizzling feather shafts and the associated morphogenetic process, engineers are able to fabricate composite beams with the desired curling features. These composite beams offer compelling advantages for various applications [8]. By combining a stiff outer shell with a lightweight, porous core, the design achieves exceptional specific strength, enhanced resilience, and superior restoring capacity. Furthermore, fabricating these beams in various geometries of ridges and contours allows for tailored designs that optimize performance, integrate seamlessly into assemblies, and minimize material waste. The curly configuration, mimicking the natural bending of frizzling rachis, offers distinct benefits such as reduced stress concentrations, improved fatigue and impact resistance, and enhanced structural stability. Curly composite beams inspired by the frizzling rachis are ideal for applications demanding lightweight yet robust components, spanning aerospace structures, automotive chassis, architectural elements, sporting equipment, and assistive devices.

In conclusion, our study offers an in-depth understanding of the morphological modifications of the cortex–medulla composite in feather rachis across scales under various frizzle allele expressions. By elucidating the complex interplay between genetics and the microstructure, we uncover how Aves developed the composite feather shaft, exhibiting unique material and morphological characteristics. Moreover, our analytical approach holds promise for exploring the diverse morphological variations observed in feathers across different bird species, shedding light on the emulation of the keratin-based construction for various functionalities [13,14].

## Data Availability

The authors confirm that the data supporting the findings of this study are available within the article. The MATLAB codes utilized to support this study have been deposited to Mendeley Data: https://doi.org/10.17632/v33v6ykccb.1 (accessed on 3 June 2024).
